# Influence of Spirituality and Modesty on Acceptance of Self-Sampling for Cervical Cancer Screening

**DOI:** 10.1371/journal.pone.0141679

**Published:** 2015-11-03

**Authors:** Eileen O. Dareng, Elima Jedy-Agba, Patience Bamisaye, Fatima Isa Modibbo, Lawal O. Oyeneyin, Ayodele S. Adewole, Olayinka B. Olaniyan, Patrick S. Dakum, Paul D. Pharoah, Clement A. Adebamowo

**Affiliations:** 1 Centre for Cancer Genetic Epidemiology, Department of Public Health and Primary Care, University of Cambridge, Cambridge, United Kingdom; 2 Institute of Human Virology Nigeria (IHVN), Abuja, Nigeria; 3 Department of Non-communicable Disease Epidemiology, London School of Hygiene and Tropical Medicine, London, United Kingdom; 4 Department of Nursing Services, National Hospital, Abuja, Nigeria; 5 Department of Medical Microbiology and Parasitology, National Hospital, Abuja, Nigeria; 6 Mother and Child Hospital Ondo, Ondo, Nigeria; 7 Department of Obstetrics and Gynaecology, National Hospital, Abuja, Nigeria; 8 Department of Epidemiology and Public Health; Institute of Human Virology and Greenebaum Cancer Centre, School of Medicine, University of Maryland, Baltimore, Maryland, United States of America; Rudjer Boskovic Institute, CROATIA

## Abstract

**Introduction:**

Whereas systematic screening programs have reduced the incidence of cervical cancer in developed countries, the incidence remains high in developing countries. Among several barriers to uptake of cervical cancer screening, the roles of religious and cultural factors such as modesty have been poorly studied. Knowledge about these factors is important because of the potential to overcome them using strategies such as self-collection of cervico-vaginal samples. In this study we evaluate the influence of spirituality and modesty on the acceptance of self-sampling for cervical cancer screening.

**Methodology:**

We enrolled 600 participants in Nigeria between August and October 2014 and collected information on spirituality and modesty using two scales. We used principal component analysis to extract scores for spirituality and modesty and logistic regression models to evaluate the association between spirituality, modesty and preference for self-sampling. All analyses were performed using STATA 12 (Stata Corporation, College Station, Texas, USA).

**Results:**

Some 581 (97%) women had complete data for analysis. Most (69%) were married, 50% were Christian and 44% were from the south western part of Nigeria. Overall, 19% (110/581) of the women preferred self-sampling to being sampled by a health care provider. Adjusting for age and socioeconomic status, spirituality, religious affiliation and geographic location were significantly associated with preference for self-sampling, while modesty was not significantly associated. The multivariable OR (95% CI, p-value) for association with self-sampling were 0.88 (0.78–0.99, 0.03) for spirituality, 1.69 (1.09–2.64, 0.02) for religious affiliation and 0.96 (0.86–1.08, 0.51) for modesty.

**Conclusion:**

Our results show the importance of taking cultural and religious beliefs and practices into consideration in planning health interventions like cervical cancer screening. To succeed, public health interventions and the education to promote it must be related to the target population and its preferences.

## Introduction

In contrast to many developed countries, the burden of cervical cancer has remained high in most developing countries [1–3]. Of the 528 000 new cervical cancer cases that occurred globally in 2012, 85% of these were in Sub Saharan Africa [2]. This is largely due to the absence of large scale population based screening programs [4]. In these countries, cervical cancer screening efforts are largely opportunistic and sporadic, resulting in low uptake and lack of public health benefit [4].

Several methods of cervical cancer screening have been utilized since the introduction of Pap smear in 1928 [5]. More recently, HPV DNA testing is increasingly used with several countries implementing it as a primary screening method, as a result of a greater protection against invasive cancer, when compared to cytology [6–8]. This is largely because HPV DNA based testing is more objective and more reproducible compared to other cervical cancer screening modalities [9–11]. Considering that the infrastructural requirements for HPV DNA based testing is less stringent than required for cytology based screening, with fewer clinic visits required, this may be a feasible alternative in resource limited environments [9].

In order to substantially reduce the burden of cervical cancer in Sub Saharan Africa, there is a need to implement sustainable and accessible population based screening programs and address the barriers to cervical cancer screening uptake. Several studies have explored some of these barriers. These have focused on socioeconomic barriers (cost of screening, stigma associated with a positive result, embarrassment, lack of trust in the health care systems, spousal disapproval); health systems barriers (accessibility, multiple visits, discomfort, complications from screening procedure, sex of health care providers) and lack of awareness. Few have examined the role of religious and cultural factors [4, 12–16]

There are several challenges in evaluating the effects of religiosity and health outcomes. One of these is the difficulty in defining the concept of religiosity and understanding it as a measurable trait that influences health care behaviour [17–19]. Till date, most studies on health outcome and religiosity in Sub Saharan Africa have typically used one general measure, such as attendance at religious gatherings or active religious participation or observance, without recognizing the multidimensional nature of this attribute [20]. Furthermore the use of a singular measure such as attendance at religious gatherings may be heavily confounded. For example, increase in religious participation may serve as a coping mechanism in dealing with illness and conversely illnesses that result in reduced mobility may reduce religious participation [21]. Despite the difficulties in measurement, studies show that religions help shape the way individuals interpret and seek help for their illnesses [22]. It is therefore important to evaluate the influence of religiosity in interventions to improve health care utilization.

Self-collected cervical samples for HPV DNA based tests for cervical cancer screening may reduce some of the barriers to uptake including those related to religious and cultural norms of modesty, fear of pelvic examinations, concerns about the sex of the health care provider, sanitary environment of the health care centers and requirements for multiple clinic visits [23]. Although several studies have indicated that the use of self-sampling methods for HPV DNA detection is feasible in African countries, few studies have evaluated its acceptability and even fewer have explored the potential barriers to its use, particularly the influence of spirituality and modesty in communities with high levels of self-reported spirituality [11, 20, 24–27]. In this study we evaluated the influence of self-reported spirituality and modesty on the uptake of cervical cancer screening, in particular how these affect acceptance of self-collected samples, in order to inform the design of targeted interventions that would increase cervical cancer screening uptake in these communities.

## Methods

### Study Population

Between August and October 2014, we recruited 600 women in Ondo and Abuja in the south western and north central regions of Nigeria respectively. Women were eligible to participate if they were over 18 years with no obvious physical ailments. We spoke to eligible women at markets, mosques, churches, schools, banks, and on the streets and invited them to participate. The authors EOD, EJA, PB and FIM led the recruitment team and were assisted by TU, CO, SI, EO, FA, OO and OA (acknowledged in the paper). All recruiters were females, familiar with local cultures and norms and dressed in culturally appropriate attire during recruitment. Participants had the options of a self-administered questionnaire or an interviewer administered questionnaire. We obtained written informed consent from all women before they were enrolled in the study.

We collected demographic information including age, education, religion, ethnicity and occupation. In order to evaluate socioeconomic status we asked about household ownership of consumer goods (car, motorcycle, refrigerator, television, bicycle and fan), characteristics of the household dwelling (type of toilet facility, separate room for cooking, source of fuel for cooking, source of drinking water and type of residence) and house ownership. We also collected information on knowledge of cancer and of cancer screening, and attitudes towards screening for any cancer in general, and specifically for cervical cancer. Preference for self-sampling was collected as a categorical variable with four possible responses (prefer to self-sample at home, prefer health care provider irrespective of gender in a hospital, prefer female health care worker in a hospital, and prefer male healthcare worker in a hospital) For this analysis responses were dichotomized into two—prefer to self-sample at home and prefer a health care provider in the hospital.

### Measurement of spirituality

To measure spirituality, we used a 7 item mini-scale derived from the Brief Multidimensional Measure of Religiousness/Spirituality (B-MMRS) (Table 1) [18]. We asked participants to rate the items on a 5 point Likert scale ranging from strongly disagree to strongly agree.

**Table 1:** Mini scales for the measurement of spirituality and modesty, Nigeria, 2014.

Spirituality mini-scale ^a^	Modesty mini-scale ^f^
I feel God’s presence every day and I am comforted by his presence ^b^	I admit my faults and I apologize when my mistakes are pointed out to me
I find strength and comfort in my religion most of the time ^b^	I have a high opinion of myself and confidence in my ability to handle most situations that arise
I believe that God watches over me at all times ^c^	I believe that I should dress conservatively at all times
I feel a deep sense of responsibility in reducing pain and suffering in the world ^c^	My religion dictates that I dress conservatively at all times
The events in my life happen according to God’s predestined plan for me ^d^	When working in a team, I am happy to take the lead most times
How frequently do you engage in prayers to God ^e^	I do not like drawing attention to myself
How frequently do you attend religious gatherings ^e^	When addressing conflicts with others, I prefer to be tactful and inoffensive
	I like to receive praise for my work

^a^ derived from the Brief Multidimensional Measure of Religiousness/Spirituality (B-MMRS)

^b^ From the Daily Spiritual Experiences domain

^c^ From the Values/Beliefs domain

^d^ From the Meaning domain

^e^ From the Religious Practices Domain. Responses to these questions were in 5 categories of Never, Once in 3 months, Once a month, Twice a Month and At least once a Month

^f^ derived from definitions of modesty by Gregg *et al*

### Measurement of modesty

To assess modesty, we developed an 8-item mini scale (Table 1) from definitions of modesty by Gregg *et al*, which was based on surveying what people generally understand by the term modesty [28]. Participants were asked to respond to the statements in the mini-scales on a 5 point Likert scale ranging from strongly disagree to strongly agree.

### Statistical analysis

We examined knowledge of cervical cancer using 3 variables: ever heard of cancer, ever heard of cervical cancer and knowledge of at least one symptom of cervical cancer. We analyzed continuous and categorical variables using the Wilcoxon rank sum test and Pearson’s chi squared test or Fischer’s exact test respectively.

#### Spirituality analysis

In order to identify the variable, spirituality, we analysed data from the 7 item mini-scale using Principal Component Analysis (PCA) [29]. We evaluated sampling adequacy and suitability of our data for PCA by computing the Kaiser-Meyer-Olkin (KMO) values, conducting the Bartlett’s test of sphericity, and examination of the correlation matrix of our variables [30–32]. The items “frequency of prayers and attendance at religious gatherings were poorly correlated with other variables in the 7 item spirituality mini scale (highest correlation was 0.16). Furthermore, the KMO values for “frequency of prayers” and “attendance at religious gatherings” were 0.53 and 0.50 respectively, which are both considered “miserable” for KMO scores [32]. Therefore these items were excluded. Sensitivity analysis showed that the inclusion of the poorly correlated variables: frequency of prayers and attendance at religious gatherings in the spirituality score did not significantly alter the results with a p-value of 0.04 for the goodness of fit test. After exclusion, all KMO values for the individual items were ≥0.80 and the overall KMO measure was 0.83. The Bartlett’s test of sphericity showed a patterned relationship between the items (χ2 (6) = 1513.35, p< 0.001). We used the multiple criteria of an eigenvalue cut-off of 1, cumulative variance explained, scree plot and interpretability of extracted components to determine the number of components to retain [33–35]. We identified one component that explained the cumulative variance of 70%. We predicted scores using the component loadings for the retained component and each participant received a score for the variable spirituality. Each score was a linear combination of the component loadings of the items used to measure spirituality. The variable, spirituality should be interpreted as a continuum and participants may exist at any point in this continuum.

#### Modesty analysis

We performed PCA on the 8 items collected on the modesty mini scale. Individual KMO values ranged from 0.77 to 0.87 and overall KMO measure was 0.83. Barlett’s test of sphericity showed a patterned relationship between the items χ^2^ (21) = 1566.07.49, *p*< 0.001). Using an eigenvalue cut off of 1 and examining the scree plot, we extracted one component that explained 52% of the total variance.

#### Socioeconomic Status Analysis

To estimate socioeconomic status, we calculated the wealth index using PCA of data on household assets as described by Filmer and Pritchett [36]. Categorical variables were converted to dummy variables with binary responses and principal component analysis was performed on 21 variables. The first component in the principal factor analysis explains the largest proportion of the total variance, so that assets that vary the most across participants had a larger weight and assets owned by all participants had a weight of zero. The weights for each asset for this first component was used to generate wealth scores. Based on these scores, participants were classified into 3 socioeconomic classes: the lowest 40% as low class, the middle 40% as the middle class and the top 20% as upper class.

#### Logistic Regression Models

We used logistic regression models to study the associations between spirituality and modesty, and preference for self-sampling. We ran 2 separate models: one in which the predictor of interest was spirituality and in the other, the predictor of interest was modesty. Variables that were associated with preference for self-sampling in age-adjusted analyses with a p-value of ≤0.10 were included in the multivariable logistic regression models. Next we evaluated overall model fit by checking for linearity, collinearity and performing Hosmer-Lemeshow goodness of fit tests. All analyses were conducted in STATA 12 (Stata Corporation, College Station, Texas, USA).

### Ethical Consideration

The study was conducted according to the Nigerian National Code for Health Research Ethics and the Declaration of Helsinki. Ethical approval to conduct this study was obtained from National Health Research Ethics Committee of Nigeria (NHREC/01/01/2007–01/08/2014) and all participants provided written informed consent using consent forms and procedures approved by the National Health Research Ethics Committee of Nigeria.

## Results

Some 630 women were approached to participate and 30 (5%) declined citing time constraints. We did not directly observe any differences in characteristics such as dress, language or behaviour among women who declined to participate. Of the 600 women who participated, 19 women had missing spirituality and modesty mini-scales’ data resulting in 581 participants for whom complete data was available and are included in this analysis. Table 2 describes the demographics, social characteristics and cervical cancer knowledge of participants. Most participants were married (70%), employed (76%) and had some form of formal education (94%). Participants’ religious affiliations were roughly evenly split between Muslims (50%) and Christians (50%). Most participants (89%) had heard about cancer, however relatively fewer participants were aware of cervical cancer (43%) or its symptoms (33%). Overall 19% (110/581) of participants preferred self-sampling to sampling by a health care provider.

**Table 2:** Demographic, social characteristics and cervical cancer knowledge of participants by sampling preference, Nigeria, 2014.

Characteristic	Sampling Preference			
	Total N = 581	Health care provider N = 471	Self-sampling N = 110	*p* ^a^
Age (years) Median (IQR)^b^	29 (12)	30 (5)	28 (10)	0.01
Occupation N (%)				0.19
Unemployed	137 (23.6)	110 (23.4)	27 (24.6)	
Professional	78 (13.4)	66 (14.0)	12 (10.9)	
Managerial	69 (11.9)	52 (11.0)	17 (15.5)	
Non manual skilled	78 (13.4)	69 (14.7)	9 (8.2)	
Manual skilled	190 (32.7)	148 (31.4)	42 (38.2)	
Semi-skilled/Unskilled	29 (5.0)	26 (5.5)	3 (2.7)	
Religion N (%)				0.03
Christian	292 (50.3)	247 (52.4)	45 (40.9)	
Muslim	289 (49.7)	224 (47.6)	65 (59.1)	
Geographical location N (%)				<0.001
South west	254 (43.7)	184 (39.1)	70 (63.6)	
North central	327 (56.3)	286 (60.9)	40 (36.4)	
Marital status N (%)				0.09
Married	405 (69.7)	321 (68.2)	84 (76.4)	
Not Married ^c^	176 ((30.3)	150 (31.8)	26 (23.6)	
Highest educational level N (%)				0.12
No formal	37 (6.4)	31 (6.6)	6 (5.4)	
Primary (6 years)	95 (16.3)	71 (15.1)	24 (21.8)	
Secondary (12 years)	191 (32.9)	149 (31.6)	42 (38.2)	
Tertiary (16 years)	163 (28.1)	141 (29.9)	22 (20.0)	
Post graduate (≥ 17 years)	95 (16.3)	79 (16.8)	16 (14.6)	
Ever heard of cancer N (%)	516 (88.8)	427 (90.7)	89 (80.9)	0.01
Ever heard of cervical cancer N (%)	249 (42.9)	206 (43.7)	43 (39.1)	0.38
Awareness of one or more cervical cancer symptoms N (%)	189 (32.5)	161 (34.2)	28 (2.5)	0.08
Ever screened for cervical cancer N (%)	71 (12.2)	57 (12.1)	14 (12.7)	0.86
Socioeconomic status				0.002
Low	232 (39.9)	174 (36.9)	58 (52.7)	
Middle	232 (39.9)	191 (40.6)	41 (37.3)	
Upper	117 (20.2)	106 (22.5)	11 (10.0)	

Abbreviations: *p*—P value

^a^ Wilcoxon rank sum test for age modelled as a continuous variable, Fischer’s exact test is reported for age categories and occupation, Pearson’s chi2 test is reported for religion, geographical location of respondents, marital status, highest educational level, ever heard of cancer, ever heard of cervical cancer, aware of ≥ 1 cervical cancer symptom and ever screened for cervical cancer

^b^ 14 participants were missing age

^c^ This category includes participants who are single, widowed, separated or divorced. Categories were collapsed as there were very few participants who were widowed (17), separated (4) or divorced (4).

With respect to the indicators used to measure spirituality, responses were skewed. Over 90% of participants agreed or strongly agreed with the statements: I feel God’s presence every day and I am comforted by his presence; I find strength and comfort in my religion most of the time; I believe that God watches over me at all times; and I believe that the events in my life happen according to God’s predestined plan. Therefore the responses to these questions were dichotomized into 2 categories: strongly agree or not.

In multivariable analysis, we found that women who reported high levels of spirituality were less likely to prefer self-sampling (OR = 0.88, 95% CI = 0.78–0.99, p value = 0.03) adjusting for age, religious affiliation, geographic location and socioeconomic status (Table 3). Muslim women in our study were more likely to prefer self-sampling compared to Christians and the multivariable OR was 1.69 (95% CI = 1.09–2.64, p value = 0.02). Geographic location was also independently associated with preference for self-sampling with women in north central Nigeria less likely to prefer self-sampling compared to women in the south western region of the country, adjusting for age, spirituality, religious affiliation and socioeconomic status. The odds ratio was 0.47 (95% CI = 0.29–0.74, p-value = 0.001). We also found lower preference for self-sampling among women in the higher compared to lower socioeconomic class. The multivariable OR, adjusting for spirituality, age, religious affiliation and geographic location was 0.45 (95% CI = 0.41, 0.97, p value = 0.04)

**Table 3:** Association between spirituality and modesty, and preference for self-sampling: age adjusted and multivariable logistic regression models, Nigeria, 2014.

Characteristic	Univariate		Multivariable logistic regression models			
			Spirituality model		Modesty model	
	OR (CI)	*p*	OR (CI)	*p*	OR (CI)	*p*
Spirituality	0.81 (0.72, 0.90)	<0.001	0.88 (0.78, 0.99)	0.03	-	
Modesty	0.91 (0.82, 1.02)	0.12	-		0.96 (0.86, 1.08)	0.51
Age	0.97 (0.95, 1.00)	0.03	0.98 (0.96, 1.01)	0.21	0.99 (0.96, 1.01)	0.26
Religious affiliation		0.03		0.02		0.02
Christian (reference)	-		-		-	
Muslim	1.59 (1.05, 2.43)		1.69 (1.09, 2.64)		1.71 (1.10, 2.66)	
Geographic location		<0.001		0.001		<0.001
South west (reference)	-		-		-	
North central	0.37 (0.24, 0.56)		0.47 (0.29, 0.74)		0.42 (0.27, 0.68)	
Socioeconomic status						
Low (reference)	-		-		-	
Middle	0.64 (0.41, 1.01)1	0.06	0.77 (0.48, 1.24)	0.29	0.77 (0.48, 1.23)	0.27
Upper	0.31 (0.15, 0.62)	0.001	0.45 (0.41, 0.97)	0.04	0.42 (0.20, 0.89)	0.02

Abbreviations: OR—Odds Ratio, CI—Confidence Interval, *p*—P value

Modesty was not significantly associated with preference for self-sampling (OR = 0.96, 95% CI = 0.86–1.08, p value = 0.51).

## Discussion

In this study of women in Nigeria, we found an association between spirituality, religious affiliation, socio-economic status and geographical location, and self-reported preference for self-sampling for cervical cancer screening.

Women who were classified as having high levels of spirituality were less likely to report that they would accept self-sampling for cervical cancer screening. This finding may reflect perceptions of body image and a particular sensitivity about performing intra-cavitary procedures on themselves among these women. Our finding that Muslims were more likely to prefer self-sampling than Christians, which was not attenuated after adjusting for spirituality, modesty, age, geographic location and socioeconomic status, may suggests that there are other cultural factors, other than spirituality, that influences the acceptance of self sampling among Muslims. From our previous qualitative research, we identified that Muslim women perceived a sense of discrimination at health care facilities which prevents them from seeking care for non-critical services (manuscript under review). This may explain the preference for self-sampling since this does not require clinic visits. Alternatively cultural practices that normalise intra-cavitary insertions may be more prevalent among Muslim women resulting in their being more comfortable with self-collecting a sample for cervical cancer screening. Studies in Uganda have also shown that women who reported inserting herbs to widen the birth canal during pregnancy or regularly douche were more comfortable with self-collection of samples (13). Acceptance of self-collection may also reflect the attitude of these women to exposure of their bodies to other people, concern about sex of health care workers and beliefs about disease aetiology.

Our finding differs from results of some studies evaluating the influence of spirituality and religion on acceptance of self-sampling. In a study of 300 women in Uganda and 50 (mostly migrant) African-Caribbean women in the UK, researchers did not identify an association between religious beliefs and self-reported preference for self-sampling. While we used several measures to ascertain religiosity, these other studies asked a single question about whether religious beliefs would influence choice of self-sampling [20, 37]. This may explain the difference in our results. Other possible explanations for the difference include variations in pattern and practice of religions and spirituality in different communities.

Participants from the south western region of Nigeria were more likely to prefer self-sampling than women from the north central region. This may reflect the higher levels of exposure to formal education and Western influence on norms and values in the south western region of the country compared to the northern region [38], as well as a higher degree of social disinhibition. Studies of African immigrants to the US suggest that the uptake of self sampling for cervical cancer screening increases with acculturation using length of residency as a proxy [39].

Overall the self-reported acceptance of self-sampling for cervical cancer prevention in this population was low. This could be partly explained by our finding that although a large proportion of participants had heard about cancer, relatively fewer participants were aware of cervical cancer, its symptoms, cervical cancer prevention/screening methods and the possibility of self-collection of samples for screening. The lack of knowledge about cervical cancer may also be related to the hidden anatomical location of the cervix in contrast to the breast, for example, which is located on the surface, as well as cultural and social inhibitions in discussing sexual and reproductive health. Although studies done in developed countries show a high level of acceptance [40], studies in Africa have revealed varying levels of acceptance of self-collection of samples for cervical cancer screening [20, 24–26, 41, 42]. Typically, studies that reported high levels of acceptance have included an educational intervention where information was provided on cervical cancer, prevention methods and self-collection of samples; evaluated acceptance after women had been given the opportunity to use the self-collection device or were conducted in women who had been screened for HPV [24, 25, 27]. In scenarios where acceptability was evaluated prior to any intervention, low acceptance rates as reported in our study was observed [41].

One major criticism of studies on religiosity is the methodological flaws in the ascertainment of spirituality using one indicator such as attendance at religious gatherings [43]. Spirituality is a complex construct that includes a wide variety of behavioural aspects, such as attendance at religious gatherings, solitary prayer, meditation, reading sacred texts, as well as attitudinal aspects such as values, beliefs and feelings [18]. We address this by measuring spirituality with a multidimensional self-report survey instrument derived from the B-MMRS taking into account four domains of religiosity—daily spiritual experiences, values and beliefs, meaning and religious practices. The B-MMRS is designed for use in heterogeneous populations (regardless of religious affiliations) among adults of all ages [18].

One of the strengths of our study is the sample size. The traditional protocol of sample size determination is power analysis, however this approach is not very useful when dealing with psychometric measurements [44]. There are varying opinions on sample size recommendations for studies utilizing data reduction techniques, with sample sizes varying from 50 [44] to 1000 [45]. The general rule of thumb of 100 as poor, 200 as fair, 300 as good, 500 as very good and 1000 or more as excellent from the work of Comrey and Lee is widely used [45]. With a sample size of 600, our study is sufficiently powered to evaluate the latent constructs of spirituality and modesty among Nigerian women.

## Conclusion

Our findings suggest that acceptability of self-sampling for cervical cancer prevention without prior educational intervention would be low among Nigerian women. Our results demonstrate the importance of taking cultural and religious beliefs and practices into consideration in planning health interventions like cervical cancer screening. In order for these to succeed, the choice of intervention and public education to promote them must be guided by adequate knowledge of the target population and its preferences in order to maximize impact. Therefore public health interventions targeted at reducing the burden of cervical cancer in developing countries need to incorporate public education on cervical cancer and its prevention.
